# Higher cognitive load interferes with head-hand coordination: virtual reality-based study

**DOI:** 10.1038/s41598-023-43337-x

**Published:** 2023-10-17

**Authors:** Adi Lustig, Meytal Wilf, Israel Dudkiewicz, Meir Plotnik

**Affiliations:** 1 Center of Advanced Technologies in Rehabilitation, Sheba Medical Center, Ramat Gan, Israel; 2 Department of Physiology and Pharmacology, Faculty of Medicine, Tel Aviv University, Tel Aviv, Israel; 3 Division of Rehabilitation, Sheba Medical Center, Ramat Gan, Israel; 4 Faculty of Medicine, Tel Aviv University, Tel Aviv, Israel; 5 Sagol School of Neuroscience, Tel Aviv University, Tel Aviv, Israel

**Keywords:** Cognitive ageing, Attention, Cognitive control, Motor control, Decision

## Abstract

Daily life activities often involve decision-based reaching movements in different contexts and circumstances. These activities span a wide array of cognitive load types we face while executing motor functions. Here we use a virtual reality-based neurocognitive testing platform to assess cognitive-induced changes in motor behavior as reflected by modulations in head-hand coordination. Our paradigm is based on the Color Trails Test (CTT), which is designed to assess two types of cognitive functions: *Trails A*—sustained visual attention (SVA), and *Trails B*—divided attention (DA). The virtual reality CTT adaptation (VR-CTT) requires execution of large multi-directional hand movements and head rotations. We employed a cross-correlation analysis on hand and head kinematics data collected from 122 healthy participants (ages: 20–90 years; divided as follows: young, middle-aged, and older adults) who completed the VR-CTT. The level of spatial coherence of head-hand movements was found to be high (R ≥ 0.76) in both Trails A and B, in all age groups. However, assessing head-hand phase shifts revealed longer time lags (i.e., in which head leads hand) in Trails B versus Trails A, in all age groups. We conclude that allocating cognitive resources to DA task reduces head-hand synchrony as compared to SVA conditions.

## Introduction

Most natural tasks require the continuous and coordinated spatiotemporal movement of the eye (i.e. saccade), head (i.e. gaze shifts), and hand (i.e. reach, grasp etc.), over various functions and during multiple sub-actions^1,2^. Specifically, the eyes, head, and hand work in a task-depended synergic sequence, which allows to assemble sufficient sensory input regarding the target/motor goal, update it with the changing perspective of the performed task, and while providing optimal visual guidance to the executing effector^1,3,4^. Within this coordinated structure, recent studies done both in humans and nonhuman primates, suggest a stronger spatiotemporal coupling of the head and the hand with respect to the eye which is more reactive in motion^1,2,5,6^. Furthermore, not only motor goals influence eye-head-hand coordination patterns. Cognition also plays an important role, since a synergy between cognitive processes and subsequent motor execution is needed for any purposeful movement (e.g., visual scanning of the environment to identify relevant objects, decisions on movement, planning hand trajectories etc.). Specifically, the coordination is mediated by top-down executive control related to the type of the task, and by the bottom-up incoming sensory stimuli interpretation from the visual scene and updated task status^7,8^. The interaction between cognitive processes and motor acts is usually studied with only one type of motor task, e.g., locomotion^9^ or arm movements^10–12^. However, the way in which cognitive load impacts coordinated movement of multiple effectors has not been studied yet.

The paradigm in which cognitive-motor interactions are being examined also has a critical influence on the subsequent behavior. Typical experimental settings require a single movement onto a straightforward or an in-filed-of-view located target which suddenly appears (i.e., flashed stimuli)^2,5,13,14^. Nevertheless, in real life, ‘targets’ do not normally appear suddenly out of thin air, but are mostly rather stable (i.e., real life objects are simply there), allowing for continuous motor planning and the use of spatial memory. Testing paradigm which includes continuous targets is, once again in this context, more comparable to the required timing and coordination of the eye-head-hand movements in real-life tasks^1,15^.

The Color Trails Test (CTT) is a well-validated version of the Trail Making Test (TMT), which assesses executive functioning, attention and processing speed, in a manually performed task using pencil-and-paper^16–18^. The CTT consists of two parts: *Trails A*—which evaluates sustained visual attention (SVA), in which participants are required to connect 25 circled numbered targets from a single set in numeric sequence (targets are colored as follows: odd-numbers are pink; even-numbers are yellow); and *Trails B*- which requires divided attention (DA), which contains 2 sets of targets (all the numbers appear in both colors), and participants are required to alternate between the two color groups while maintaining sequencing (i.e., moving from pink ball #1 to yellow ball #2 to pink ball #3 and so forth). The added divided attention component of searching for the correct target color as well as for the correct number in Trails B and the inexplicit requirement to avoid distractors (i.e., targets with the same number but different color background) consequently form a task of higher cognitive demand^19^. Trails B essentially contains the same task as Trails A, with the addition of another subtask (now also attend the color) requiring sustained and divided attention. Scoring is based on the time needed to complete the tasks, with shorter time reflecting better performance^18,20,21^.

The simplistic motor action required from the participant in the pencil-and-paper CTT (i.e., drawing a connective line between targets), and the conditions under which it is carried out (i.e., the cognitive psychologists' clinics), limit the test’s comparability with the complex demands of real-life conditions. In particular, it falls short of describing the integrative, cognitive-motor functioning which is inherent to daily function^22,23^, in common with other traditional cognitive evaluations^24–26^. The low ecological validity of the pencil-and-paper CTT format in addition to the growing evidence that VR-based neuropsychological tests are more predictive of 'functional behavior'^27–29^, led us to translate and adapt the CTT task to a VR-based platform (VR-CTT)^30–32^, consequently forming a validated and better-suited three-dimensional (3D) environment to assess cognitive-motor interactions (please see supplementary file, section A: video S1, describing the different tasks).

The VR-CTT requires greater multi-directional hand movements to reach the targets, and pronounced head rotations (i.e., around all three axes, yaw, pitch, and roll) to scan the much-spread virtual target array. In the process, the participant identifies where in space the next correct target in sequence is and generates a reaching movement to virtually touch it, by controlling a virtual effector representing the hand. The VR-CTT allows more elaborate motor actions, which are more analogous with natural daily functions. Furthermore, the digitalized VR platform enables to collect quantitative 3D kinematics data, process distinct hand and head motions, and characterize interrelations between gross manual and head scanning functions, and cognitive performance.

In this work we study head-hand motor coordination in the context of executive function by means of the VR-CTT, which allows better representation of daily function, in a highly controlled setup. Due to evidence of growing temporal asynchrony and an increase in variability of motor behavioral performance with aging^33–36^, we tested three age groups, namely, young adults (YA), middle aged adults (MA) and older adults (OA). We hypothesized that the more difficult DA task (i.e., as transpires from higher completion times) will disengage head-hand coordination as compared to pure SVA task in a tested group of healthy participants. Furthermore, we aim to study whether this effect varies with aging.

## Methods

**Figure 1:**
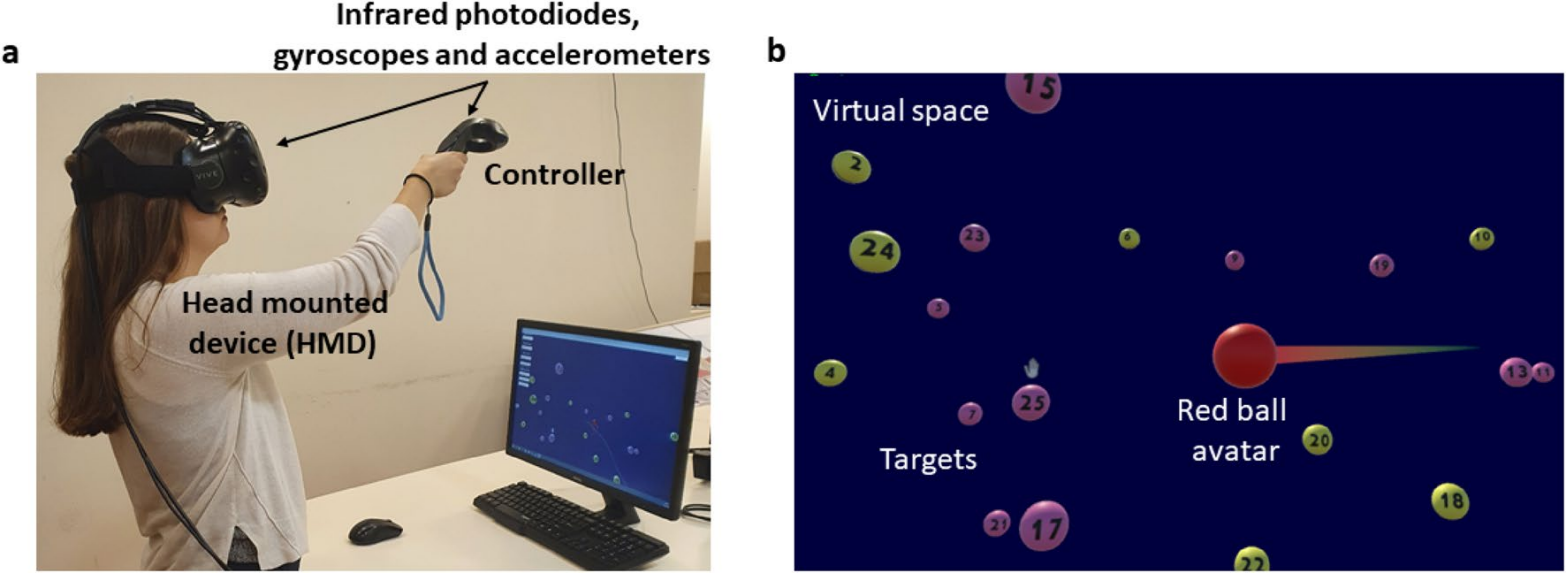
Fully immersive virtual reality-based color trails test platform: consists of a head mounted device (HMD) and a controller which includes gyroscopes, accelerometers, and infrared photodiodes through which rotational data and position are recorded (**a**). The visual scenes of Trails A and B include ball-shaped colored and numbered targets and a red ball avatar representing the participant’s hand (**b**). This image was taken for demonstration purposes only, the presenter is not a participant of the study.

In this study we further analyzed previously collected data of participants performing the VR-based CTT tasks (VR-CTT), which was recently developed and validated^32^, employing a fully immersive VR platform. A comprehensive platform design is described in detail elsewhere^32^. Briefly, the experimental set-up included a head mounted device (HMD; HTC-Vive; New Taipei City, Taiwan) and a controller by which the participant interacted with the virtual environment. The headset had a field of view (FOV) of approximately 100° and 110° in the horizontal and vertical axes, respectively. A gyroscope and an accelerometer component located at the base units of the controller and headset, allow continuous positional tracking of the devices. Infrared photodiodes located on the surface of the headset and the controller, recover the position and the orientation for correction of drift in real-time. The spatial accuracy of the system is approximately 0.75 mm^37,38^ and the temporal accuracy is approximately 4 ms in continuous motions^39^. The synchronization between the real-world position of the hand and its corresponding position in the virtual environment enabled the manipulation of a virtual (red ball) avatar used for travelling between the target balls at the participant’s will (i.e., by manipulating the controller in space). Elements of the experimental system are shown in Fig. 1.

The visual scene of Trails A included a single set of 25 ball-shaped, colored, and numbered targets (odd-numbers are pink; even-numbers are yellow), scattered in the 3D virtual space. The participants were asked to 'hit' them using the virtual (red ball) avatar in an ascending order. In Trails B visual scene, there were two sets of ball-shaped numbered targets in two colors (i.e., each number appeared in both yellow and pink, corresponding to standard CTT) and participants were required to alternate between ball colors while tracing the number sequence. Prior to performing each of the VR-CTT parts, acclimation practice trails were conducted to confirm that participants can clearly see target balls and the numbers labeling them, maneuver the avatar ball by the controller, and are aware of the spatial dimensions of the virtual arena where target balls can be located. At this stage participants were also introduced to the positive feedback of a correct 'hit' (i.e., visual cue; momentary inflation of the target ball to twice its regular size). Lastly, before starting a task, participants were made aware that their execution time is being recorded and that in the case of an error a visual and auditory notice will be given and they will need to return to the correct target ball before proceeding.

### Participants

We analyzed data collected from 122 healthy participants categorized into three age groups: young adults (YA) ages 18–39 years (n = 40); middle-aged adults (MA) ages 40–64 years (n = 51); and older adults (OA) ages 65–90 years (n = 31). One OA participant (age: 70 years) could not perform the VR-CTT practice and actual test levels, showing general disorientation. Another participant from this group (age: 89 years) asked to stop the VR-CTT during Trails B, expressing frustration at his perceived poor performance. Other than these incidents no difficulties were reported, including motion sickness. Power analyses based on effect sizes (Partial Eta Squared, η^2^) were used to determine a sufficient sample size for investigation. To achieve a medium effect size (i.e., η^2^ = 0.5) with alpha of 5% and power of 80%, a minimum of 31 participants in each of the study groups (i.e., YA, MA, and OA) were required (using Gpower^40^). Specifically, the analysis described in this work was focused on the middle-aged adults group chosen as a representative sample and was ultimately expanded to show generalizability of the findings in the other two age groups. Inclusion criteria were able-bodied participants, males and females in the ages of 18–90. Exclusion criteria were motor, balance, psychiatric or cognitive conditions that may hinder understanding of the instructions or completing the tasks. Additionally, qualified participants were required to self-report having vision acuity 20/20 (or corrected to 20/20) and no color blindness which may affect target identification. Notably, the colors of the CTT were chosen to allow also color-blind subjects to distinguish them as dark (i.e., pink) and light (i.e., yellow)^18^. To assure this later criterion, at the beginning of the experimental session subjects were asked to name the color and the number of target balls presented in a different arrangement than those presented in the trials. The experimental protocol was approved by the Sheba Medical Center institutional review board (IRB). All participants signed a written informed consent prior to enrolling to the study. All methods and procedures used in this study were performed in accordance with the relevant guidelines and regulations.

### Data analyses and outcome measures

**Figure 2:**
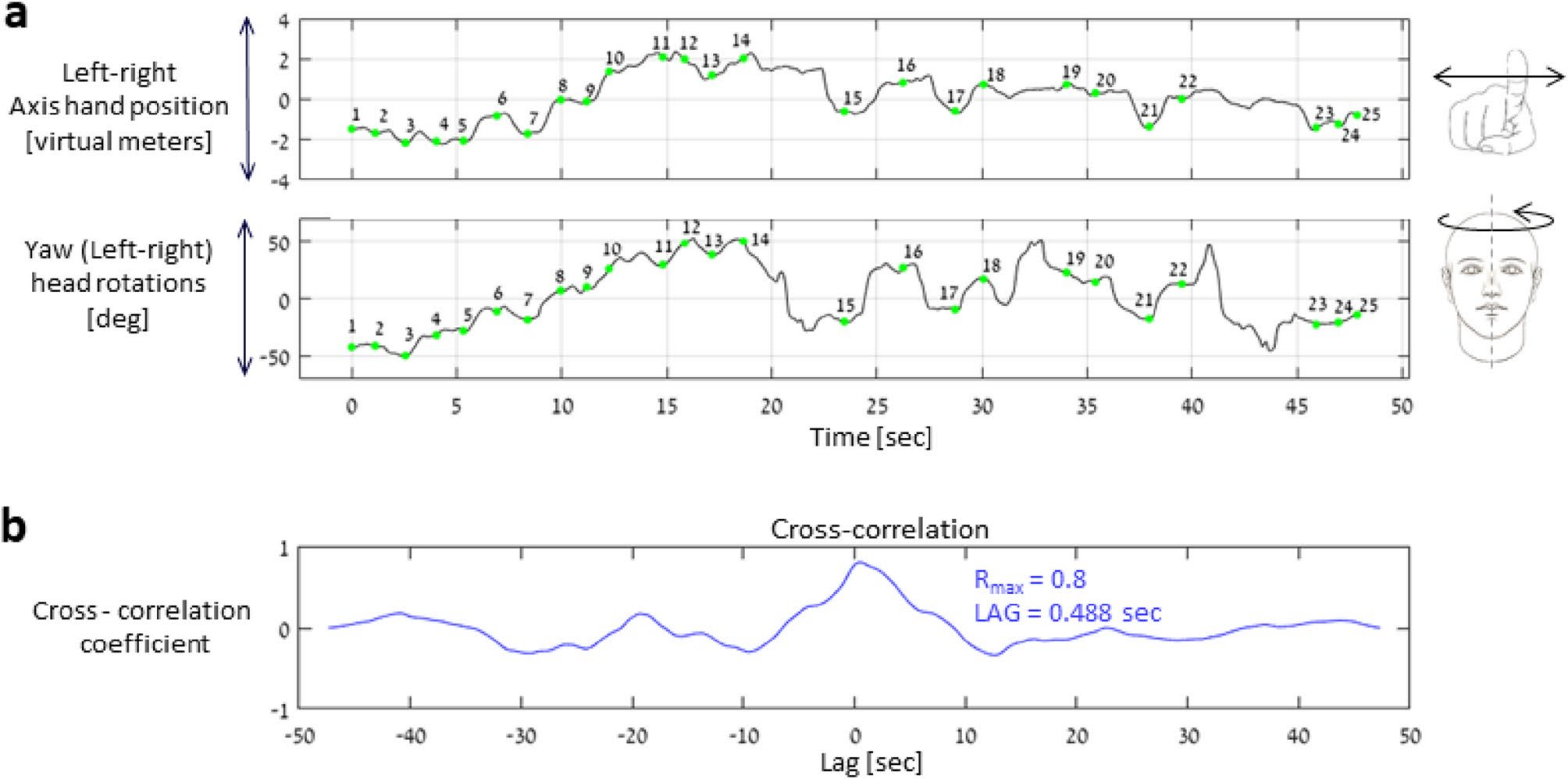
The level and characteristics of the coupled motion of the hand and the head. Examples of horizontal axis hand trajectory and head rotation yaw angles coupled motion (**a**), as well as the cross-correlation curve and the outcome measures: maximal cross-correlation coefficient (R_max_) and time lag (LAG) between head-hand motions (**b**) for one participant who performed Trails A.

The digitalized platform recorded the spatial trajectory of the participant’s hand and his/her head rotation angels (both in 3D), the recorded data were then pre-processed (i.e., spatially centered to a common coordinate system and segmented) using a MATLAB-based software and 24 target-to-target arm reaching movements and head rotations were extracted. In order to determine the level and characteristics of the coupled motion of the hand and the head, we used the cross-correlation function (MATLAB software, ver. R2020b, MathWorks, Inc., Natick, MA, USA) on the following sets of data: (i) hand horizontal trajectory profile and (ii) head rotation angel around the yaw axis (see Fig. 2a). The maximal cross-correlation coefficient (R_max_) was extracted from the best-fit configuration between the superimposed signals and the phase shift (i.e., temporal delay—LAG) between the signals was registered (Fig. 2b). R_max_ describes the spatially related coordination in generating hand movements and head rotations in the left–right plane. LAG describes to what extent the hand movement lagged the exploring head movements while locating the target to be reached (positive values of LAG). The total completion time of each participant for Trails A (t_A_) and Trails B (t_B_) was recorded, similarly to the standard practice of the pencil-and-paper CTT. Normalization across age groups of an outcome measure was performed first by grouping the resulted outcome measure of all subjects (across age groups and separately for Trails A and Trails B) and calculating the mean and standard deviation of the group. Next, a z score of the outcome measure, based on the group mean and standard deviation, was calculated to obtain a normalized scoring, across all the three age groups.

### Statistical analyses

Analysis of variance (ANOVA) was used to assess effects of Age group (YA, MA, OA; between subjects factor), Trails (Trails A, Trails B; within -subjects factor) and Age group X Trails interaction. Partial Eta Squared was computed as a measure of effect size. In pre-hoc analyses, Shapiro–Wilk normality tests were used for each outcome measure, in each age group. Of the eighteen normality tests conducted to the maximal cross-correlation coefficient (R_max)_, time lag (LAG) and completion time (t) outcome measures (2 per Trails A/B and 3 per age group), more than half of the samples indicated non-normal distributions. Accordingly, the data were log-transformed prior to applying ANOVA tests and a non-parametric approach was chosen for post-hoc statistical analysis. On the new data sets we confirmed homogeneity of variance assumption using the Levene’s test based on median for Trails A and B across Age groups (p > 0.05). Descriptive statistics, figures and correlations analyses were performed on the pre transformed data. Paired, two-tailed Wilcoxon signed rank tests for related samples were performed (on the pre transformed data) to detect a potential statistically significant Trails difference (Trails A, Trails B; within-subjects factor) in the R_max_ and LAG measures, separately for each age group (YA, MA, OA). The resulted p-values were adjusted with the Bonferroni correction. The Kruskal–Wallis test was used to evaluate statistically significant differences across Age group (YA, MA, OA; between-subjects factor) in the R_max_ and LAG measures, on each of the Trails separately (Trails A, Trails B). A post-hoc Dunn's multiple comparisons, to identify any specific significant differences between the age groups were then performed. The resulted p-values were adjusted with the Bonferroni correction. The Spearman correlation coefficient (r_s_) was used to quantify association between parameters or normalized parameters. In post hoc analyses, linear relations were assessed using Pearson’s correlation analyses. The statistical significance level was set as *p* < 0.05. Statistical analyses were run using SPSS software (SPSS Ver. 27, IBM).

## Results

In total, we analyzed 244 full sequences (i.e., 24 consecutive segments from target #1 to target #25) of hand trajectories and head rotations from 122 participants who completed Trails A and Trails B of the VR-CTT. Firstly, the statistical analysis of the current study confirms previously discovered trends of completion time outcome measures^32^, having a main effect of Trails (F_2,119_ = 350.49, p < 0.0001, η^2^ = 0.75; longer completion times in Trails B versus Trails A; see Table 1) and Age group (F_2,119_ = 41.95, p < 0.0001, η^2^ = 0.41; longer completion times with aging; see Table 1).

**Table 1:** Calculated outcome measures for all tested age groups.

		Young adults		Middle-aged adults		Old adults	
		Trails A	Trails B	Trails A	Trails B	Trails A	Trails B
Spatial similarity	R_max_	0.77 ± 0.07	0.76 ± 0.09	0.77 ± 0.11^ϯ^	0.77 ± 0.08^ϯ^	0.77 ± 0.08	0.78 ± 0.09
Temporal delay	LAG (s)	0.42 ± 0.34	0.78 ± 0.58	0.55 ± 0.35^ϯ^	0.97 ± 0.57^ϯ^	0.69 ± 0.41	1.09 ± 0.80
Completion time	t (s)	71.17 ± 16.93	117.25 ± 25.54	89.08 ± 30.47	154.55 ± 48.48	126.05 ± 54.00	225.87 ± 81.78
Correlation of spatial similarity and temporal delay	r_s_	r_s_ = − 0.17	r_s_ = − 0.42	r_s_ = − 0.42	r_s_ = − 0.33	r_s_ = − 0.35	r_s_ = − 0.38
		*p* = 0.2810	*p* = 0.0072	*p* = 0.0017	*p* = 0.0148	*p* = 0.0496^¥^	*p* = 0.0371^¥^
Correlation of spatial similarity and completion time	r_s_	r_s_ = − 0.38	r_s_ = − 0.32	r_s_ = − 0.59^§^	r_s_ = − 0.33^§^	r_s_ = 0.08	r_s_ = 0.07
		*p* = 0.0171	*p* = 0.0423^¥^	*p* < 0.0001	*p* = 0.0169	*p* = 0.6542	*p* = 0.7100

^ϯ^See Fig. 3 for additional description of data.

^¥^Above Bonferroni correction values (0.025).

Additional description of data is shown in supplementary file, sections B, C, D: Figs. S1–S8.

As for the outcomes at focus in the present study (i.e., LAG and R_max_), statistical analysis revealed a main effect of Trails (F_2,119_ = 17.12, p < 0.0001, η^2^ = 0.12; higher LAG values, i.e., head movements precede hand movements in Trails B versus Trails A) and Age group (F_2,119_ = 5.28, p = 0.006, η^2^ = 0.08; higher LAG values with aging) for the LAG measure. No effects of Trails (F_1,120_ = 0.07, p = 0.791, η^2^ = 0.001) nor Age group (F_1,120_ = 0.304, p = 0.739, η^2^ = 0.001) were found for the R_max_ measure. None of the Age group X Trails interactions were found significant (p > 0.142).

### Spatially and temporally coordinated movements of the head and hand

**Figure 3:**
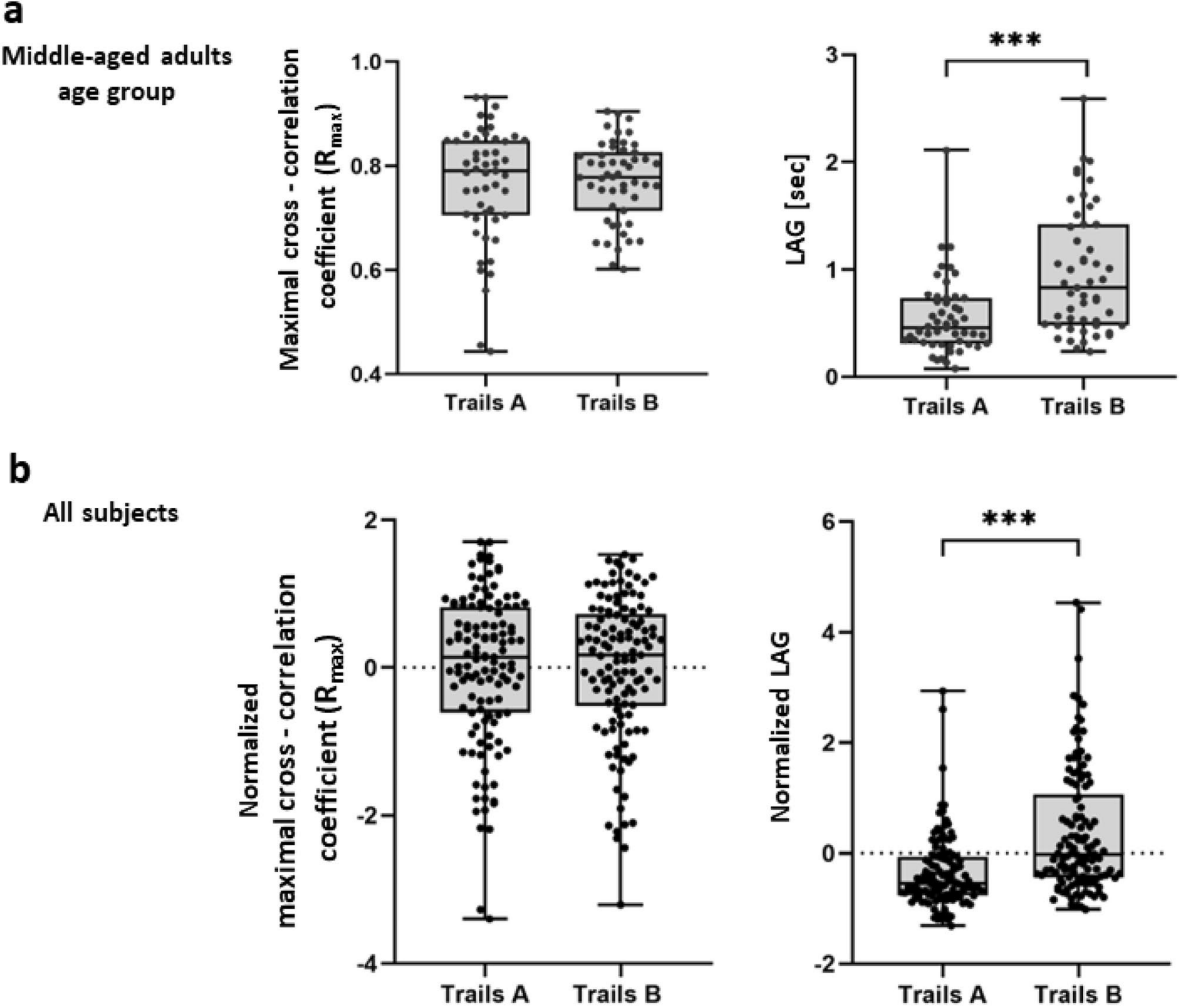
Spatially and temporally coordinated movement of the head and hand during the performance of VR-CTT. Maximal cross-correlation coefficient (Rmax; left panel) and time lag (LAG; right panel) between head-hand motions of the middle-aged adults group (N = 51) (**a**), and of all participants across age groups (N = 122) (**b**). For combining data in panel (**b**), values from each age group were 'Z-scored', i.e., dashed line (0), represents mean value of all data points in the panel. Asterisks indicate a statistically significant difference between a pair of means (p < 0.0001).

The level of spatial similarity between the hand and the head motions was found to be relatively high in both Trails A and B, as demonstrated by the resulted values of the maximal cross-correlation coefficient (R_max_) of MA group (N = 51; Fig. 3a, left panel) as follows: 0.77 ± 0.11 (mean ± SD) for Trails A, and 0.77 ± 0.07 for Trails B. The phase shift (LAG) between the hand and the head movements, extracted from the best-fit configuration of the superimposed signals, revealed longer LAG values in Trails B compared to Trails A, i.e., 0.97 ± 0.57 s (mean ± SD) for Trails A vs. 0.55 ± 0.35 s in Trails B in the MA group (N = 51, *p* < 0.0001; Fig. 3a, right panel). Similar maximal cross-correlation coefficient (R_max_) and phase shift (LAG) results were found in a normalized analysis (across age groups) of all participants (N = 122, *p* < 0.0001; Fig. 3b), as well as for a separate analysis of YA and OA age groups (see supplementary file, sections B: Figs. S1 and S2, respectively).

### Correlation of spatial motion similarity index (R_max_) and temporal delay (LAG)

**Figure 4:**
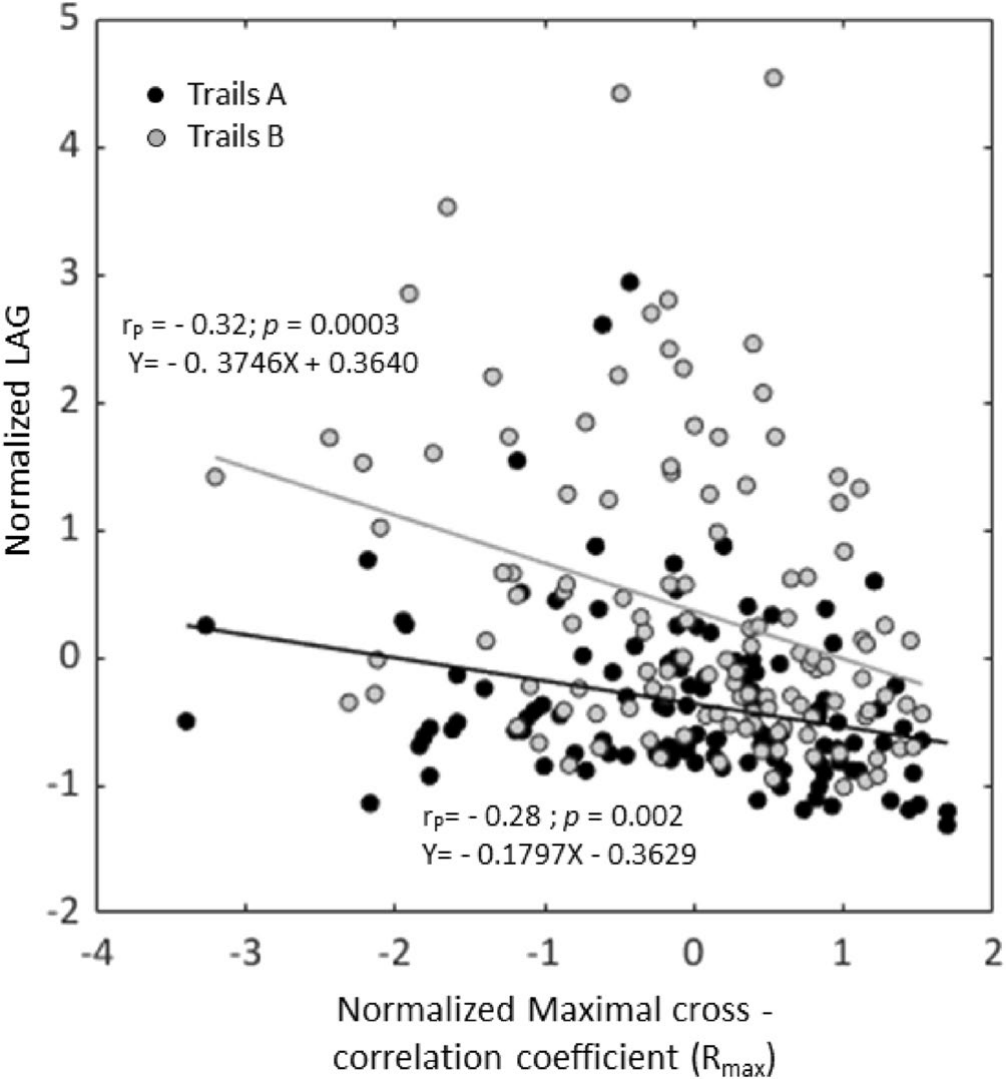
Correlation of the normalized (across age group) spatial motion similarity index (R_max_) and temporal delay (LAG). Spatial motion similarity normalized score plotted against the corresponding normalized score of temporal delay (LAG) for participants of all age groups, for Trails A (black) and Trails B (gray). Pearson correlations coefficients (r_p_) are shown for Trails A and Trails B, and regression lines are plotted.

Spatial similarity of hand and head movements (R_max_) was found to be inversely associated with the temporal delay between the head and the hand (LAG) both in Trails A (Spearman’s correlation analysis; r_s_ = − 0.35; *p* < 0.0001) and in Trails B (r_s_ = − 0.35; *p* < 0.0001, normalized LAG scoring across age groups is shown; Fig. 4), meaning that higher spatial coupling between head and hand incurred higher temporal synchrony. These associations were fitted linearly (Fig. 4). Correlation of the spatial similarity index (R_max_) with the temporal delay (LAG) of each age group separately are provided in the supplementary file, section C: Figs. S3, S4 and S5).

### Correlation of spatial motion similarity index (R_max_) and task completion time

**Figure 5:**
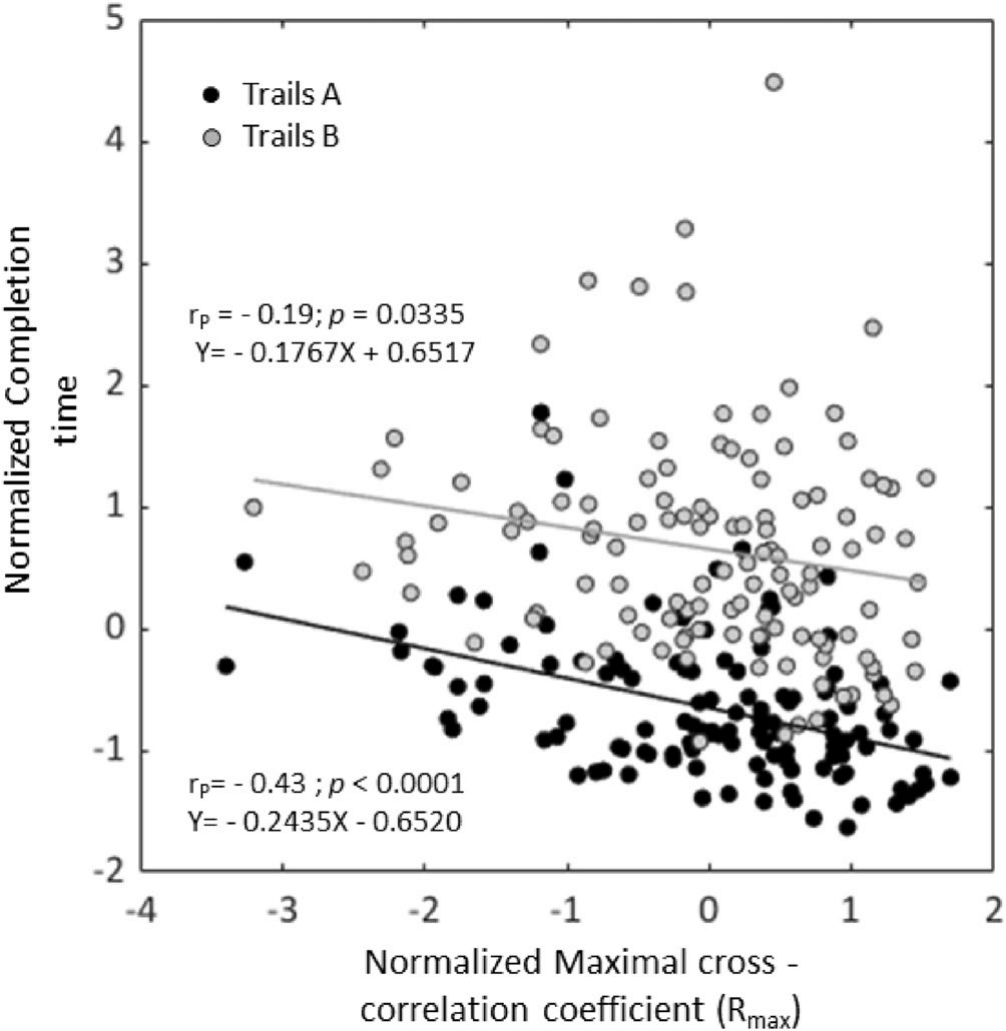
Correlation of normalized (across age groups) spatial motion similarity index (R_max_) and completion time (t): Completion times normalized scoring (t_A_—black circles, t_B_—gray circles) are plotted against the normalized spatial motion similarity index (R_max_) for participants of all age groups. Pearson correlations coefficients are shown, and regression lines are plotted for significant linear correlation (see text for Spearman’s correlation analyses).

Statistically significant inverse relation (Spearman’s analysis) was found between completion times and R_max_ for both Trails A (i.e., t_A_ vs. R_max_; r_s_ = − 0.43; *p* < 0.0001) and Trails B (i.e., t_B_ vs. R_max_; r_s_ = − 0.25; *p* = 0.0056) in a normalized analysis (across age groups) including all participants. These invers relations were fitted linearly (Fig. 5). Correlation of the spatial similarity index (R_max_) with the completion time of each age group separately are provided in the supplementary file, section D: Figs. S6, S7 and S8).

### Effect of age

**Figure 6:**
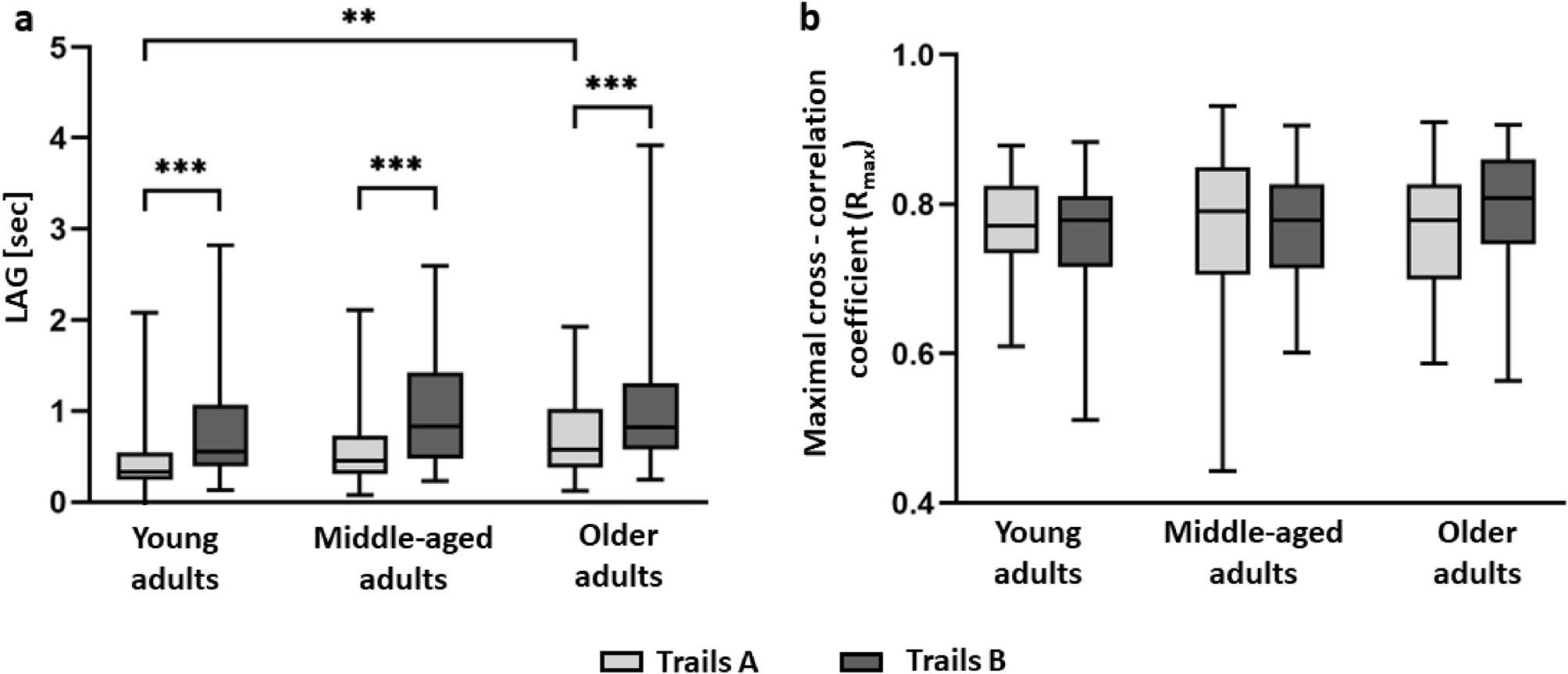
Effect of age. Significant Trails and Age group effects are reflected in increased time lags in Trails B compared to Trails A for all age groups, and in the Older adults compared to the Young adults age groups for performance of Trails A (**a**). No significant Trails or Age effect were found for the R_max_ measure (**b**).

The significant increase in temporal delay (LAG) found in Trails B as compared to Trails A (i.e., Trails effect) is observed in all age groups—namely, YA, MA and OA (*p* < 0.001, *p* < 0.001 and *p* = 0.006, respectively; Fig. 6A). No statistical difference of spatial similarity index (R_max_) between Trails A and B was documented for these age groups ( *p *≥ 0.672; Fig. 6b).

A significant Age effect was found for the LAG measure, for Trails A (*p* = 0.003; Fig. 6A), the source of which is in the dichotomic comparison between the YA and OA age groups (*p* = 0.0020; Fig. 6A). No significant Age effect was replicated for Trails B (*p* = 0.0518). Furthermore, no significant Age effect was found for the spatial similarity index (R_max_), tested for both Trails A (*p* = 0.906) and B (*p* = 0.234), see Fig. 6b. Both the difference in phase shift (LAG) across Trails, and the stable cross-correlation coefficient (R_max_) across Trails stay comparable across age groups as reflected by the fact that none of the Trails X Age group interactions were found significant (*p* > 0.142; Fig. 6).

## Discussion

In this study, we evaluated head-hand coordination in the context of cognitive task performance. For this purpose, we applied a cross correlation analysis on gross manual and head rotation kinematics collected from healthy participants, while they were performing the VR-CTT. We observed that hand movements during task performance lag behind the searching head rotations to a greater extent when DA task was involved (i.e., Trails B) as compared to SVA task (Trails A). Yet the level of similarity in trajectories patterns between head and hand movements remained comparable (i.e., similar R_max_). Former studies investigated the effects of cognitive-motor interference on postural control and stance^41^, prism adaptation (Redding et al.^42^; recently translated to VR by Wilf et al.^43^), locomotion and intra-limb coordination^44,45^, upper limbs movements^45^ and executive function in developmental deficient populations^46^. These studies provide evidence of the cost of cognitive-motor interference, leading to alterations in either cognitive or motor performance. In other studies head and hand coordination was investigated during the execution of single-leveled simple tasks^1,47^. This is the first time, to the best of our knowledge, that head-hand coordination level is being directly evaluated under different contexts of cognitive function with the use of a robust and well-known neurocognitive test.

Daily life activities often involve the need to scan the environment in continuous and coordinated movements of the eye and the head, in order to make a decision regarding the confirmed location of an object that we want to reach. In coordination with these activities the end effector is activated, i.e., the hand reaches towards the target. We encounter decision-based reaching movements in different daily contexts and circumstances e.g., reaching for different ingredients while cooking, which span a wide array of cognitive load types we face during everyday motor functions and that have specific manifestations of cognitive-motor interferences. However, experimental set-ups designed to delineate multifactorial behavior associated with cognitive-motor interactions are limited. The highly ecological, three-dimensional, VR environment (i.e., VR-CTT) utilized here is a novel paradigm to assess cognitive-induced changes in motor synergistic behavior.

The wide array of projected targets in the VR-CTT (i.e., FOV of approximately 100° and 110° in the horizontal and vertical planes, respectively), is larger than the human central visual filed (i.e., approximately 30° for both horizontal and vertical planes), in which humans focus gaze and are able to recognize and detect shapes and symbols^48,49^. In the VR-CTT platform, for any visual focal point, at any given moment, there are target balls positioned in the peripheral visual field, and thus require an active searching behavior e.g., movement of the eyes and the head from side to side and up and down, to explore and choose the correct target to aim at. Likewise, the wide spatial arrangement of the target balls requires reaching movements which vary in size, direction, and orientation. In other words, the paradigm used in this study is highly comparable to daily life behavior, i.e., scanning and reaching objects in context of cognitive function.

The level of spatial similarity between the hand and the head movements was found to be high and particularly stable across Trails A and B, and for all age groups, as reflected by the spatial similarity indices (R_max_) measured (Fig. 3a,b, Table 1, supplementary file, section B: Figs. S1a and S2a). The consistency of this outcome measure ascertains previous finings that the head and the hand are strongly coupled^1,2,5,6^. In the present study, the high head-hand spatial coupling is underscored by the fact that neither the type of the concurrent cognitive load, nor age, had an effect on this coupling.

Temporal shift outcome, i.e., LAG, revealed significantly longer time delays in which the hand lags the head in Trails B as compared to Trails A (recall Figs. 3 and 6, Table 1, supplementary file, section B: Figs. S1b and S2b). We found that during the DA condition (Trails B), which is a more demanding task as reflected by longer completion times (i.e., as compared to Trails A, see Table 1), motor activity of the reaching hand is more dissociated in time from the preceding head scanning activity, as compared to what is seen during SVA. The greater number of targets and distractors, and the dual instruction to select the correct target number and the correct color in Trails B elevates the level of required cognitive processing, which leads to strategy alterations in ‘multi-motor’ performance, as a way to overcome the related difficulty. Specifically, the cognitive process taking place in conjunction with the searching head lasts longer, therefore the motor execution of the hand is being delayed and there is a bigger lag between head and hand motor functions. Alternatively, trailing the executing hand after the head, or pausing the hand momentarily, reduces the number of variables in the system which leads to the utilization of less motor-control associated cognitive assets, releasing additional attentional resources. The latter interpretation is in agreement with the central capacity sharing model^45,50^ which suggests dual task interference is caused by a cognitive capacity limitation which requires re-distributing resources between the tasks.

Shorter temporal delay between the head and hand motions was found to be correlated to greater spatial motion similarity both in the task primarily involving SVA (Trails A) and for that involving DA (Trails B) (recall Fig. 4, Table 1). The codependency of high spatial and temporal similarities suggests a common strategy of action in which participants from all age groups prefer to keep their hand motion in close adherences to the head trajectory consequently leading to minimal time delay.

The relevance in preferring a certain motor strategy which leads to consequent alterations in behavior in the context of this study emerges from the question whether they contribute to better task performances (i.e., in terms of completion time), given the type of cognitive load. Larger spatial similarity outcomes of the head and hand were inversely correlated with completion times for both Trails A and B (recall Fig. 5, Table 1), indicating that better spatially coordinated motion between the hand and the head is associated with efficient execution of the task as reflected in shorter completion times irrespective to type of cognitive load. This outcome suggests, once again, a preferred and common strategy, which also leads to better performance of the task.

The effect of age is apparent when comparing the LAG measure for Trails A across age groups (e.g., increased temporal delay with age *p* = 0.0028; Fig. 6a). The same trend is captured for Trails B although not being statistically significant (Fig. 6). This suggests a decreased coordinated head-hand movements with ageing, and a possible distinguishable metric to differentiate among age-groups, and perhaps clinical cohorts. Furthermore, the older adults group in our study was characterized by considerably larger variability of LAG (Fig. 6) compared to other age groups (in agreement with Plotnik et al.^32^). This is in support of previous findings of growing temporal asynchrony and inconsistencies of motor behavioral performance with aging^33–36^. Further research is required to evaluate the clinical relevance of assessing the level of cognitive and motor performance in aging using the head-hand correlation metric in VR-CTT.

### Limitations, future directions, and implications

There are several limitations to our study which warrant consideration, in particular for planning future research. Firstly, we recognize that motion capture systems are generally more accurate (e.g.^51^) than positions detected from the VR system's controllers. Since our outcome is the product of spatial and temporal relation between the head rotation and hand position signals, and while assuming similar noise levels in the two signals, the related inaccuracies should have only minimal influence.

Additionally, although not being accounted for in this work, we acknowledge that eye movements have an important role in the full planning-to-execution synchronized motor behavior. Here, as a first step towards exploring integrated motor behavior of different effectors in the context of different cognitive tasks, we focused on employing the approach of correlating the motor behavior of the head and the hand. However, future studies should address eye movements' role in this kind of goal directed behavior. It should be taken into consideration that while head rotations and gross manual movement share similar end effector kinematics, eye movements are composed from saccades, smooth pursuit, and gaze fixations, and each type has its own unique kinematics.

We based our study on a widely used neurocognitive assessment which was validated as evaluating different types of cognitive demands (i.e., sustain visual attention and divided attention). Normative data of participants executing Trails B of the CTT entails longer completion time than Trails A, which is commonly interpreted as a task posing higher cognitive demands^19^. As was demonstrated in Plotnik et al.^32^, the differentiation in completion times between Trails A and B is valid also for VR-CTT across age groups. Future studies can include objective physiological measures (electroencephalography, and/or functional near-infrared spectroscopy) to demonstrate higher computational load as was previously demonstrated by Lin et al.^52^. For example, Hebbar et al.^53^, proposed that a set of physiological signals (i.e., electroencephalography, pupil diameter, gaze fixation rate, gaze distribution pattern) can be indicative, in real time, of the levels of cognitive load during task performance^53,54^.

Our study was motivated by the notion that a more holistic approach is needed to study natural human behavior. Rather than isolating the motor and cognitive competencies, these functional domains, which are constantly interacting with one another, were addressed in an integrated manner utilizing VR based paradigms. VR is emerging as a premier tool that offers the unparalleled ability to characterize and modulate, within a rigorously controlled, yet ecological, environment, the inter-relationship among human cognitive and motor systems. Understanding these interrelationships is highly important in the clinical arena where different neurological diseases (e.g., Parkinson's disease) express impairments both on motor and cognitive aspects.

## Conclusions

In the present study we revealed that the need to allocate cognitive resources for the divided attention task in Trails B, interferes with the synchronous activation of scanning head movements and gross manual movements which are engaged in the task, i.e., less synchronized head-hand motions compared to those performed in a sustained visual attention task. Taken together, these findings indicate strategic alterations in cognitive-motor integration associated with type of cognitive load, i.e., allowing longer head scanning time prior to the execution of the manual reaching movement. Our approach for characterizing the head and hand temporally and spatially coordinated movements, is part of an initial effort to develop reliable quantification methods and metrics to study multifactorial behaviors and assess cognitive-motor interactions during naturalistic behavior.

### Supplementary Information

Supplementary Video 1.

Supplementary Information 1.

## Data Availability

The datasets generated during and/or analyzed during the current study are available from the corresponding author on reasonable request.
