# Author Correction: Higher cognitive load interferes with head-hand coordination: virtual reality-based study

**DOI:** 10.1038/s41598-023-49413-6

**Published:** 2023-12-18

**Authors:** Adi Lustig, Meytal Wilf, Israel Dudkiewicz, Meir Plotnik

**Affiliations:** 1 Center of Advanced Technologies in Rehabilitation, Sheba Medical Center, Ramat Gan, Israel; 2 Department of Physiology and Pharmacology, Faculty of Medicine, Tel Aviv University, Tel Aviv, Israel; 3 Division of Rehabilitation, Sheba Medical Center, Ramat Gan, Israel; 4 Faculty of Medicine, Tel Aviv University, Tel Aviv, Israel; 5 Sagol School of Neuroscience, Tel Aviv University, Tel Aviv, Israel

Correction to: *Scientific Reports* 10.1038/s41598-023-43337-x, published online 17 October 2023

The original version of this Article contained errors in the names of the authors Adi Lustig, Meytal Wilf, Israel Dudkiewicz & Meir Plotnik, which were incorrectly given as Lustig Adi, Wilf Meytal, Dudkiewicz Israel & Plotnik Meir.

The original Article and accompanying Supplementary Information video file have been corrected.

