# Supplementary material for: Higher cognitive load interferes with head-hand coordination: virtual reality-based study
Source: Sci Rep. 2023 Oct 17;13:17632. doi: 10.1038/s41598-023-43337-x (PMC10582046; doi:10.1038/s41598-023-43337-x)
Supplement: Supplementary file 2 — Supplementary Information 1. [file 41598_2023_43337_MOESM2_ESM.docx]

1. **Supporting material for the *Introduction* section:** [**Video**](https://drive.google.com/file/d/1MKalzIDrU66Td3xnwEZnj1Kpvk7Klis1/view?usp=sharing) (https://drive.google.com/file/d/1MKalzIDrU66Td3xnwEZnj1Kpvk7Klis1/view?usp=sharing)
2. **Supporting material for the *Results* section, subsection *Spatially and Temporally Coordinated Movements of the Head and Hand*:**
3. *Young adults*


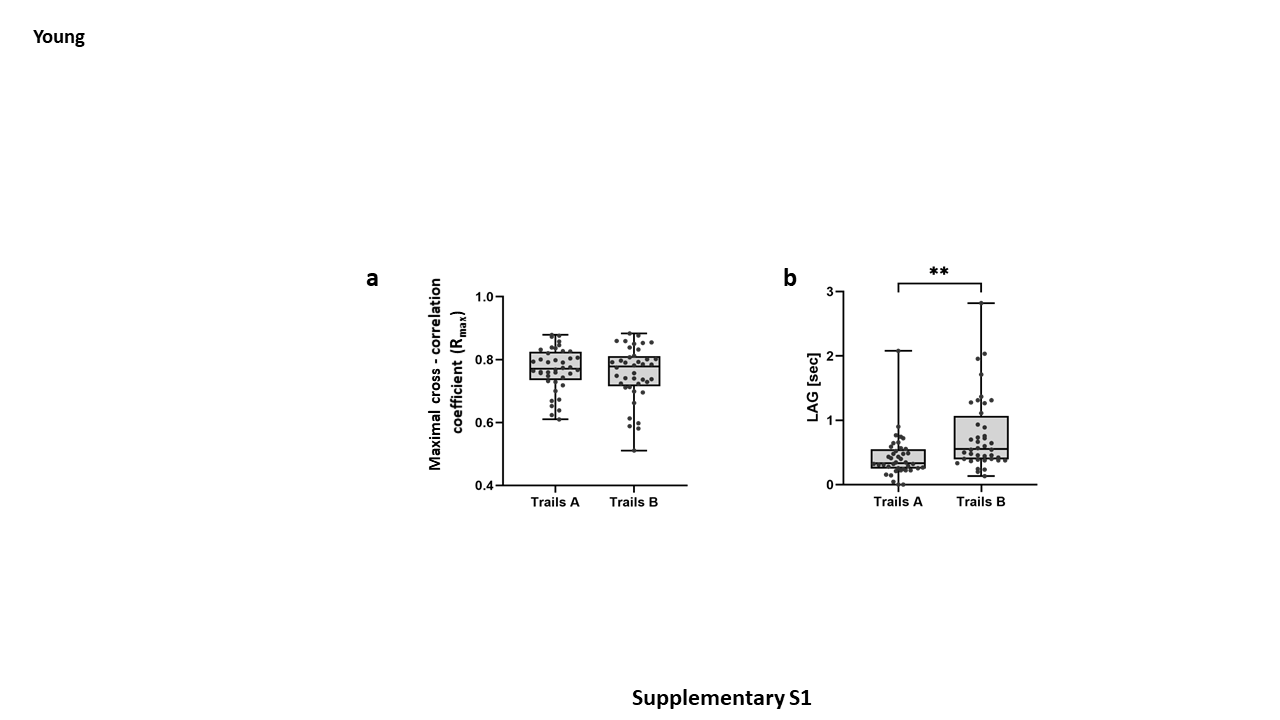
The level of spatial similarity between the hand and the head motions was found to be relatively high in both Trails A and B, as demonstrated by the resulted values of the maximal cross-correlation coefficient (R_max_) as follows: 0.77 ± 0.07 (mean ± SD) and 0.76 ± 0.09 for Trails A and Trails, respectively (N=40; Figure S1a). Longer LAG values i.e., phase shifts between the hand and the head movements were documented in Trails B compared to Trails A, i.e., 0.78 ± 0.58 seconds (mean ± SD) vs. 0.42 ± 0.34 seconds, respectively (N=40, *p*<0.0001; Figure S1b).

***Figure S1.*** *Spatially and temporally coordinated movement of the head and hand during the performance of CTT:* maximal cross-correlation coefficient (R_max_) **(a)** and time lag (LAG) between head-hand motions **(b)**. N=40 of the young adults age group (data of all participants are shown), asterisks indicate a statistically significant difference between a pair of means (p<0.0001).

1. *Older adults*


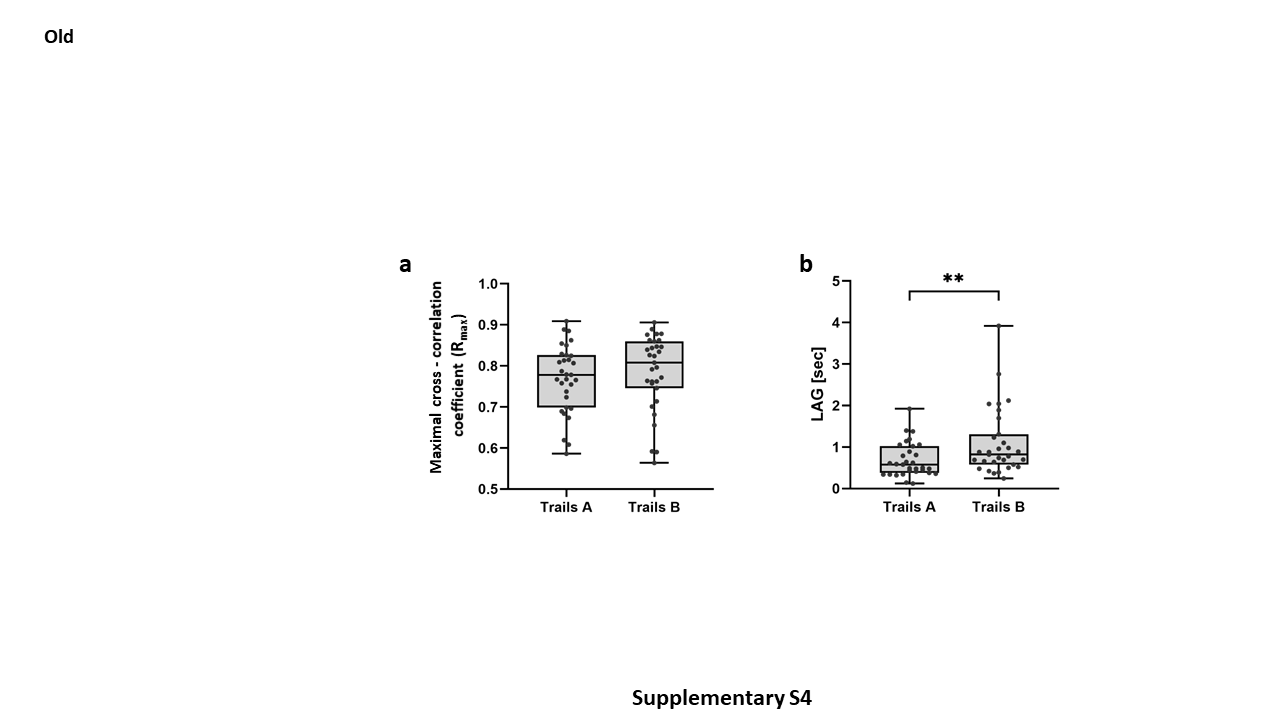
The level of spatial similarity between the hand and the head motions was found to be relatively high in both Trails A and B, reflected by the resulted values of the maximal cross-correlation coefficient (R_max_) as follows: 0.77 ± 0.08 (mean ± SD) and 0.78 ± 0.09 for Trails A and Trails, respectively (N=31; Figure S2a). Longer LAG values i.e., phase shifts between the hand and the head movements were documented in Trails B compared to Trails A, i.e., 1.09 ± 0.80 seconds (mean ± SD) vs. 0.69 ± 0.41 seconds, respectively (N=31, *p*<0.0001; Figure S2b).

***Figure S2.*** *Spatially and temporally coordinated movement of the head and hand during the performance of CTT:* maximal cross-correlation coefficient (R_max_) **(a)** and time lag (LAG) between head-hand motions **(b)**. N=31 of the older adults age group (data of all participants are shown), asterisks indicate a statistically significant difference between a pair of means (p<0.0001).

1. **Supporting material for the *Results* section, subsection *Correlation of Spatial Motion Similarity Index (R_max_) and Temporal Delay (LAG)*:**
2. *Young adults*

Spatial similarity of hand and head movements (R_max_) was found to be inversely associated with the temporal delay between the head and the hand (LAG) for Trails B (Spearman’s correlation analysis; r_s_= -0.42; *p*=0.0072). This significant interaction was not replicated for Trails A (r_s_= -0.17; *p*=0.2810;) in this age group, see Figure S3. Significant interactions were fitted linearly.


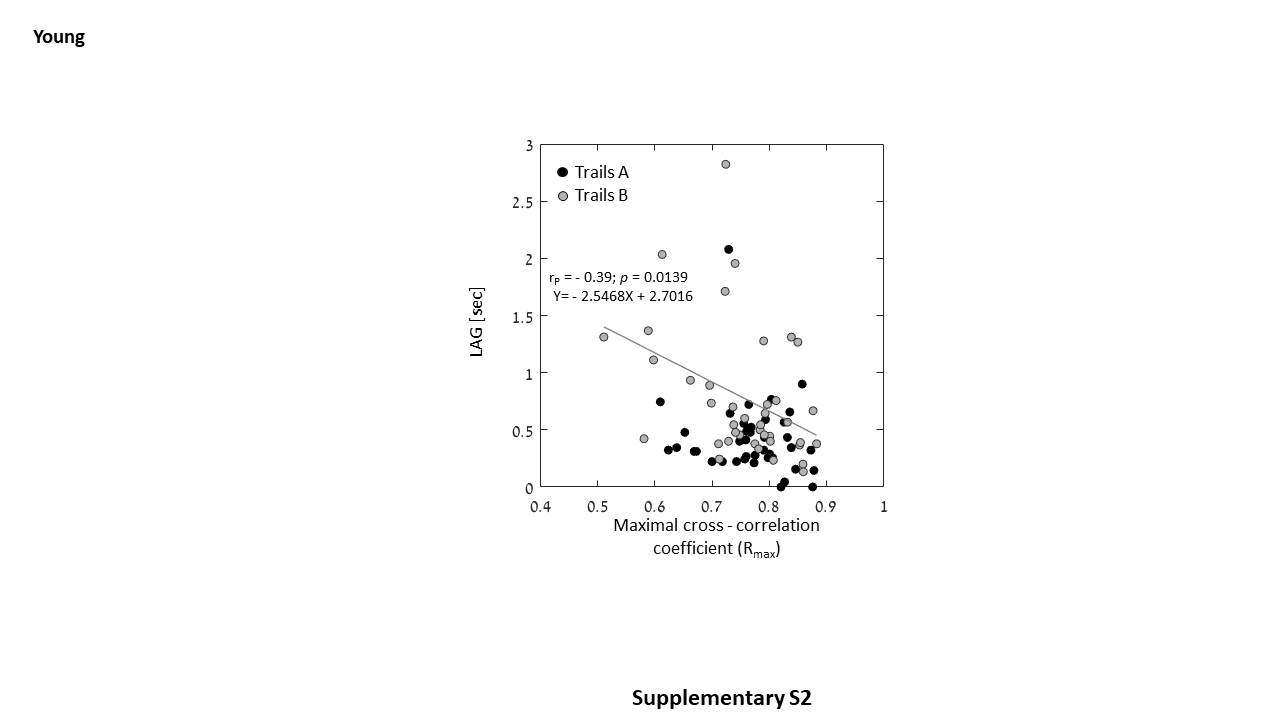


***Figure S3.*** *Correlation of spatial motion similarity index (R_max_) and temporal delay (LAG) in the young-adults age group: Spatial motion similarity index plotted against the corresponding temporal delay (LAG) for participants of the young adults age group, for Trails A (black) and Trails B (gray). Pearson correlations coefficients are shown, and regression lines are plotted for significant correlations.*

1. *Middle-aged adults*


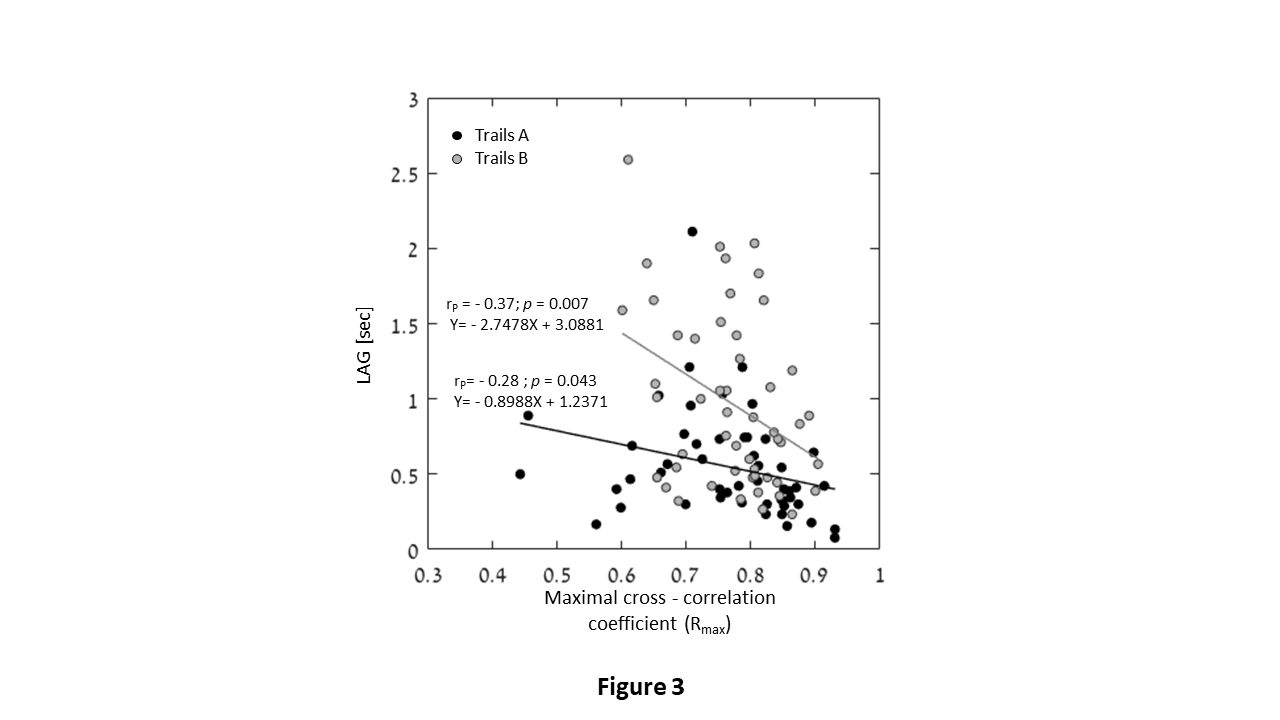
Spatial similarity of hand and head movements (R_max_) was found to be inversely associated with the temporal delay between the head and the hand (LAG) for Trails A (r_s_= -0.42; *p*=0.0017) and for Trails B (r_s_= -0.33; *p*=0.0148). These associations were fitted linearly (Figure S4).

***Figure S4.*** *Correlation of spatial motion similarity index (R_max_) and temporal delay (LAG) in the Middle-aged adults group:* Spatial motion similarity index plotted against the corresponding temporal delay (LAG) for participants of the Middle-aged adults group, for Trails A (black) and Trails B (gray). Pearson correlations coefficients are shown, and regression lines are plotted.

1. *Older adults*

Spatial similarity of hand and head movements (R_max_) was found to be inversely associated with the temporal delay between the head and the hand (LAG) for both Trails A (r_s_= -0.35; *p*=0.0496) and Trails B (r_s_= -0.38; *p*=0.0371), but only the former could be linearly fitted (see Figure S5 legend).

*
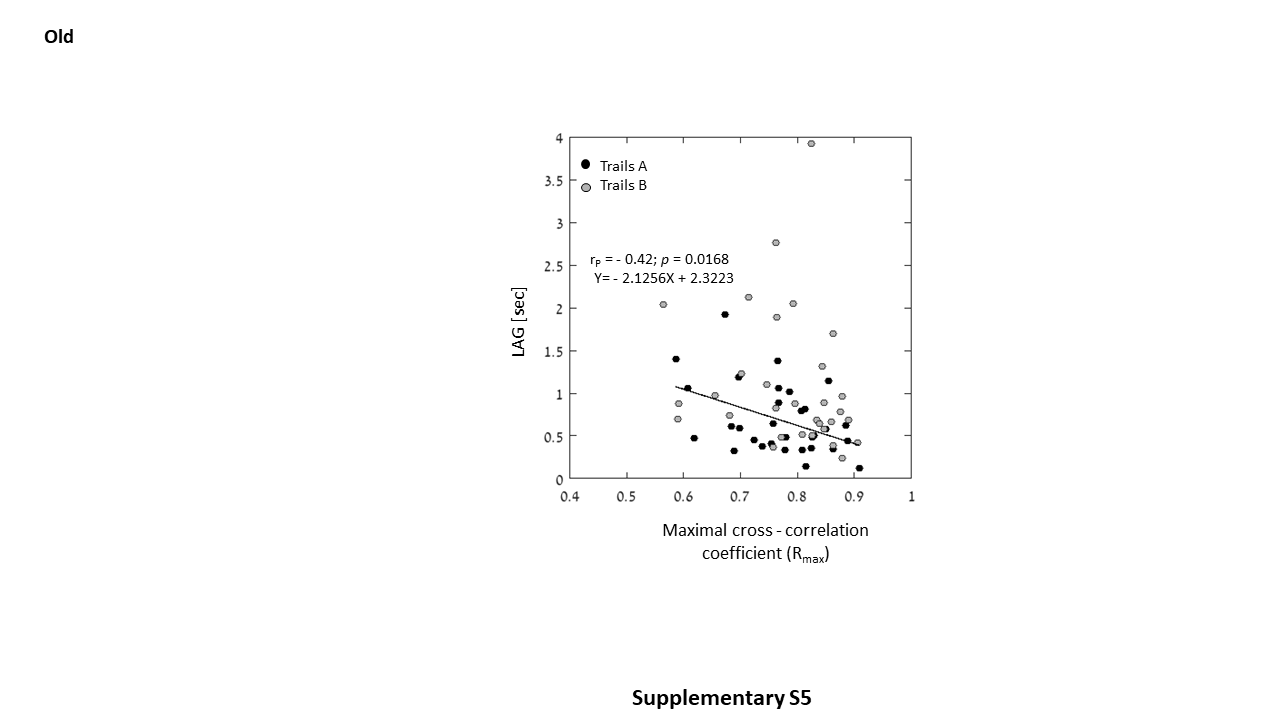
*

***Figure S5.*** *Correlation of spatial motion similarity index (R_max_) and temporal delay (LAG) for the Older adults age group:* Spatial motion similarity index plotted against the corresponding temporal delay (LAG) for participants of the older adults age group, for Trails A (black) and Trails B (gray). Pearson correlations coefficients are shown, and regression lines are plotted for significant linear correlations.

1. **Supporting material for the *Results* section, subsection *Correlation of Spatial Motion Similarity Index (Rmax) and Task Completion Time*:**
2. *Young adults*

Statistically significant inverse relation was found between completion times and R_max_ for both Trails A (i.e., t_A_ vs. R_max_; r_s_= -0.38; *p*=0.0171) and Trails B (i.e., t_B_ vs. R_max_; r_s_= -0.32; *p*=0.0423) in the young adults age group, but only the former could be linearly fitted (see Figure S3 legend).


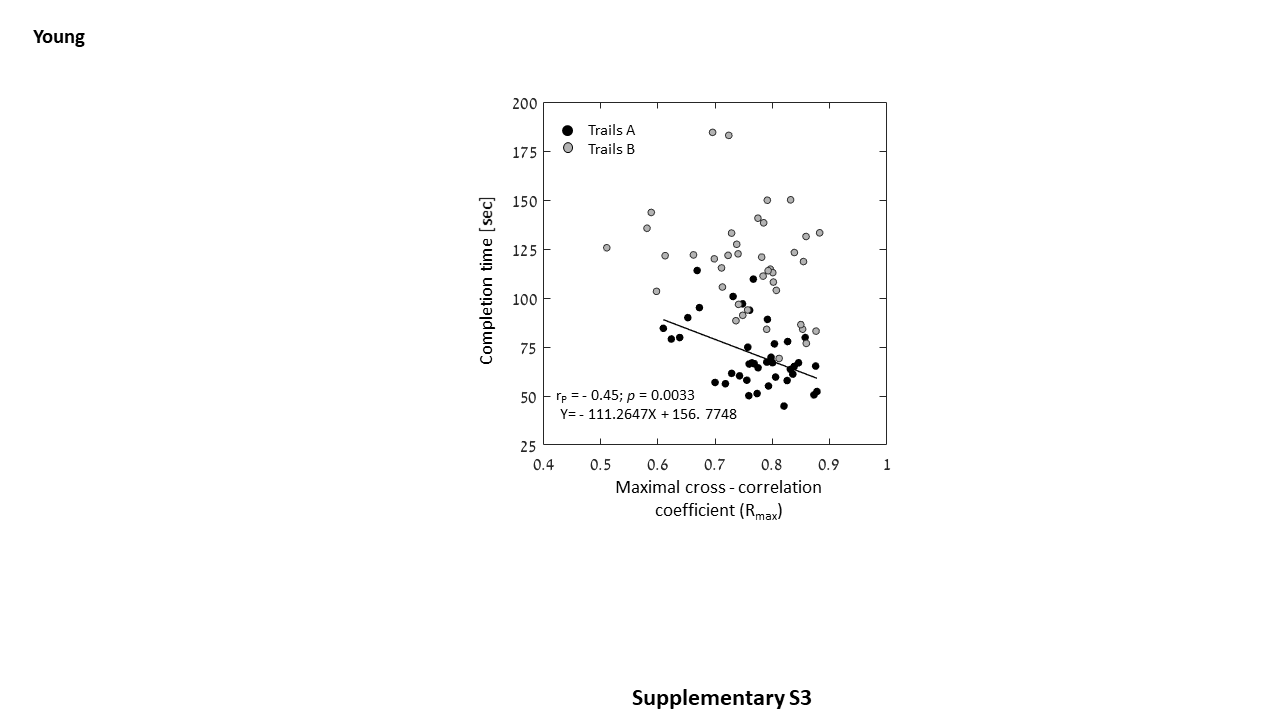


***Figure S6.*** *Correlation of spatial motion similarity index (R_max_) and completion time (t):* Spatial motion similarity index plotted against the corresponding completion time (t) for participants of the young adults age group, for Trails A (black) and Trails B (gray). Pearson correlations coefficients are shown, and regression lines are plotted for significant linear correlations.

1. *Middle- aged adults*

Statistically significant inverse relation was found between completion times and R_max_ for both Trails A (i.e., t_A_ vs. R_max_; r_s_= -0.59; *p*<0.0001) and Trails B (i.e., t_B_ vs. R_max_; r_s_= -0.33; *p*=0.0169) in this age group, but only the former could be linearly fitted (see Figure S3 legend).


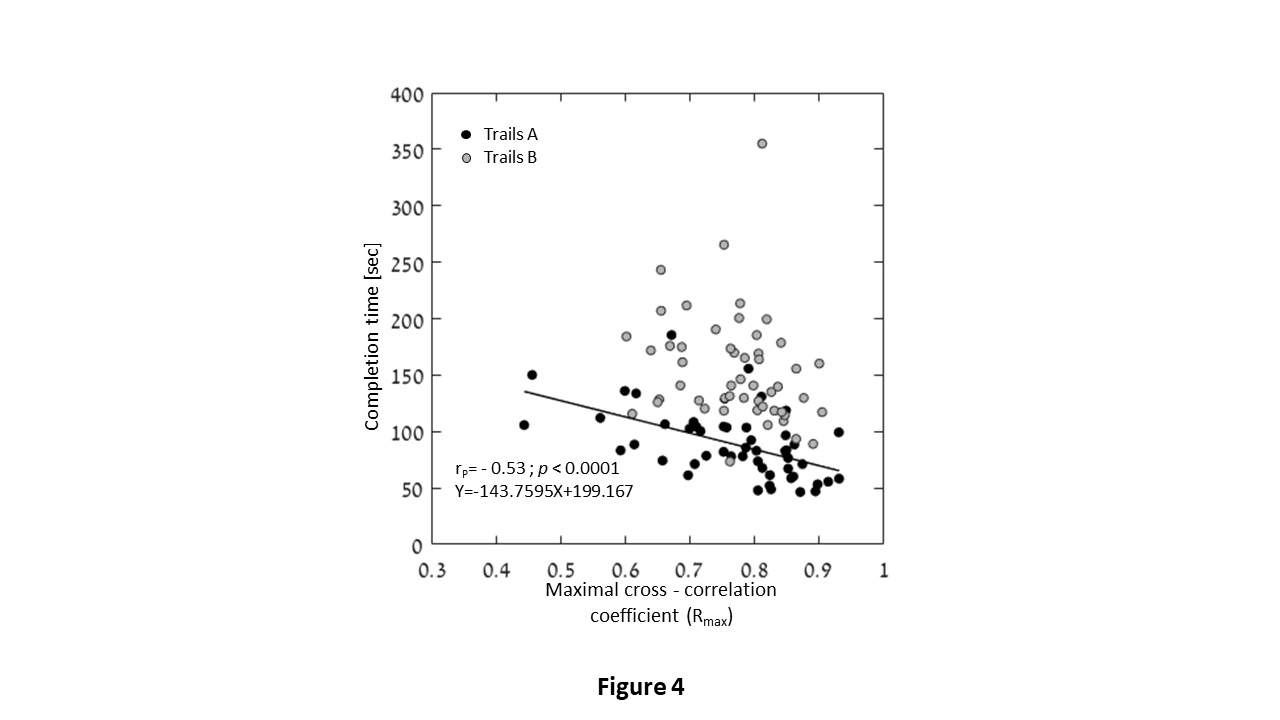


***Figure S7.*** *Correlation of spatial motion similarity index (R_max_) and completion time (t) of the Middle-aged adults group:* Spatial motion similarity index plotted against the corresponding completion time (t) for participants of the Middle-aged adults group, for Trails A (black) and Trails B (gray). Pearson correlations coefficients are shown, and regression lines are plotted for significant linear correlations.

1. *older adults*

No statistically significant relation was found between completion times and R_max_ for both Trails A (i.e., t_A_ vs. R_max_; r_s_= 0.08; *p*=0.6542) and Trails B (i.e., t_B_ vs. R_max_; r_s_= 0.07; *p*=0.7100), in the older adults age group. Suggesting this age group to have a different strategy to overcome the cognitive driven difficulty without the need to replicate the exact moving patterns of the head by the hand.


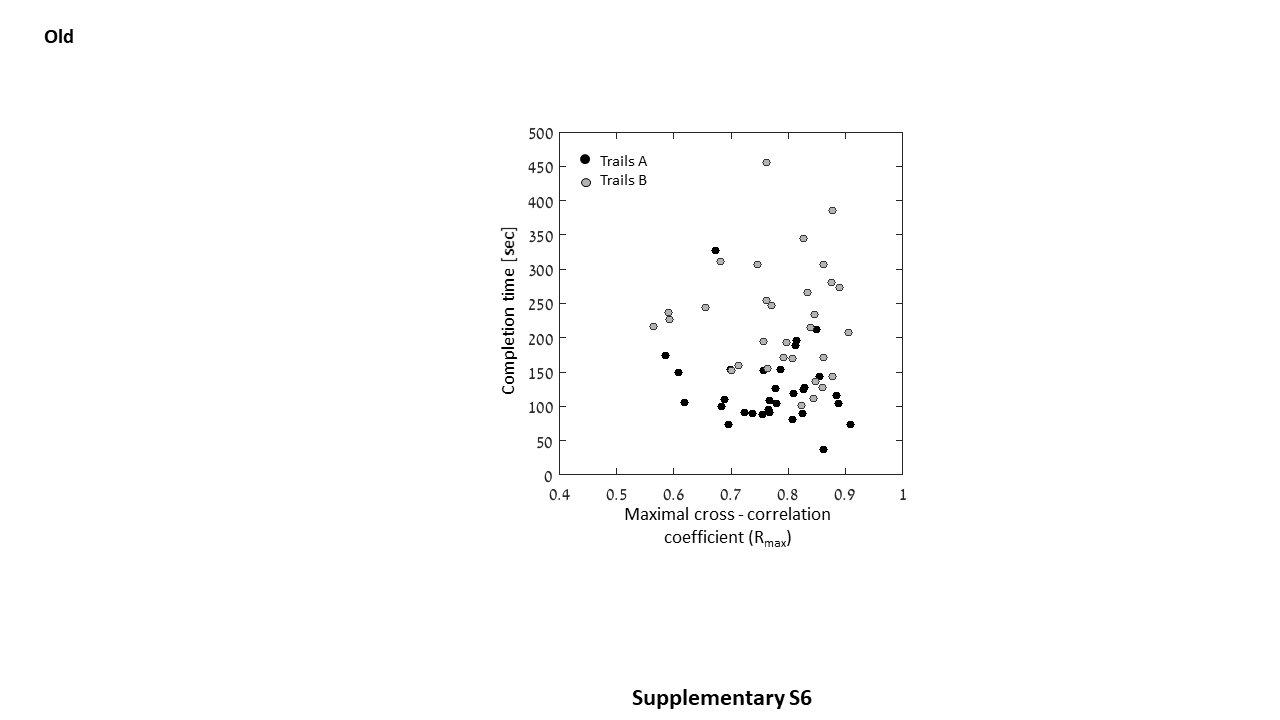


***Figure S8.*** *No correlation of spatial motion similarity index (R_max_) and completion time (t) was found for the older adults age group:* Spatial motion similarity index plotted against the corresponding completion time (t) for participants of the older adults age group, for Trails A (black) and Trails B (gray).
